# Telling Ghost Stories Around a Bonfire—A Literature Review of Acute Bleeding Secondary to Pancreatitis

**DOI:** 10.3390/medicina61010164

**Published:** 2025-01-20

**Authors:** Gabriele Bellio, Silvia Fattori, Andrea Sozzi, Matteo Maria Cimino, Hayato Kurihara

**Affiliations:** Emergency Surgery Unit, IRCCS Fondazione Ca’ Granda Ospedale Maggiore Policlinico, 20122 Milano, Italy; silvia.fattori@unimi.it (S.F.); andrea.sozzi@unimi.it (A.S.); matteo.cimino@policlinico.mi.it (M.M.C.); hayato.kurihara@policlinico.mi.it (H.K.)

**Keywords:** pancreatitis, bleeding, hemorrhage, angioembolization, surgery

## Abstract

Bleeding is a rare but serious complication of pancreatitis, significantly increasing morbidity and mortality. It can arise from various sources, including erosion of blood vessels by inflammatory processes, formation of pseudoaneurysms, and gastrointestinal bleeding. Early diagnosis and timely intervention are crucial for patient survival. Imaging modalities such as computed tomography and angiography are essential for identifying the bleeding source, where endoscopy may help in detecting and treating intraluminal hemorrhage. Management strategies for patients with extraluminal bleeding may involve angioembolization or surgical intervention, depending on the severity and location of the bleeding. While advances in diagnostic and therapeutic techniques have improved outcomes, bleeding in pancreatitis remains a challenging clinical problem requiring a multidisciplinary approach. This review aims to focus its attention specifically on the bleeding complications of pancreatitis.

## 1. Introduction

Acute pancreatitis is one of the most common complications of gallstone disease, with a reported incidence of about 5–140 cases per 100,000 [1,2].

Although biliary stones are the most frequent cause of acute pancreatitis (21–38%), other less common etiologies exist, such as alcohol consumption (16–36%), hypercalcemia, hypertriglyceridemia, iatrogenic, viral infection, and medications. However, in many cases, a clear cause is not identified [3,4].

The inflammation of the pancreatic gland has an unpredictable evolution. In fact, the pattern of possible clinical manifestations of acute pancreatitis encloses different shades of gray, going from mild cases with indolent evolution to extremely severe cases with destructive and catastrophic progression [4,5].

Although most patients (approximately 80%) do not endure an organ failure for more than 48 h (i.e., mild or moderately severe disease), 20% of cases escalate to severe disease (i.e., organ failure for more than 48 h) [4,5]. This variability justifies the mortality rate ranging from 1% to 7% in mild cases and reaching almost 20% in severe ones [4].

## 2. Methods

A review of the existing literature was carried out using PubMed and Google Scholar between September and November 2024. The terms used to research the literature were acute pancreatitis, hemorrhage, and bleeding. Case reports were not considered for inclusion. Only studies published after the year 2000 and in English were selected.

The research and selection process was conducted independently by three team members, whereas the remaining two authors checked and oversaw the study methods. The draft of the manuscript was decided, agreed upon, and reviewed by all authors.

## 3. The Bleeding Pancreatitis

### 3.1. Complications of Acute Pancreatitis

Patients affected by acute pancreatitis may suffer from different complications, both local and systemic [4].

Systemic complications include mainly single- or multiple-organ dysfunctions. Depending on the onset and duration of organ failure, acute pancreatitis can be classified as mild, moderately severe, and severe [6,7]. Defining the grading of acute pancreatitis is important because it correlates with its mortality rate: the more severe the disease, the higher the mortality [8].

The main local complications are the peripancreatic fluid collection and the necrotic fluid collection. These have the potential to evolve in about four weeks into pseudocysts and walled-off necrosis, respectively. Additionally, pancreatic necrosis may become infected, further complicating the progression of the disease [4,6].

Moreover, acute pancreatitis has a recurrence rate of 20–25%, and in about 10% of cases, it may evolve into chronic pancreatitis [9,10,11,12,13].

A peculiar group of complications of pancreatitis involves vascular complications, such as splenic and/or mesenteric vein thrombosis, disseminated intravascular coagulation, gastric varices, and pseudoaneurysm formation [14,15].

This review aims to focus its attention specifically on the bleeding complications of pancreatitis.

### 3.2. Vascular Anatomy of the Pancreas

Anatomically speaking, the pancreas is a retroperitoneal parenchymatous organ in close proximity to the duodenum on the right, the spleen on the left, and the stomach and the transverse colon on the front. Besides these, it is enclosed in a tangle of vessels, both arterials and venous, and it leans over the main branches of the portal vein and the aorta.

The arterial vascularization of the head and the uncinate process derives mainly from the pancreaticoduodenal arteries, connecting the gastroduodenal and the superior mesenteric artery. Furthermore, the body and the tail of the pancreatic gland obtain arterial blood flow from the dorsal pancreatic, greater pancreatic, and transverse pancreatic arteries, branches of the splenic artery.

The outflow involves branches of the portal system, mainly following the course of their arterial counterparts.

Even if not in direct contact with the pancreas, the aorta and the inferior vena cava run in close proximity to the gland [16,17].

### 3.3. Bleeding Complications

Bleeding complications may develop following both acute and chronic pancreatitis, and their prevalence is reported to be between 1.2% and 14.5% [18,19,20,21].

According to the literature, the arterial hemorrhagic complications of pancreatitis are mainly caused by:

- Ruptured pseudoaneurysms (60%);
- Hemorrhagic pseudocysts without pseudoaneurysms (20%);
- Capillary, venous, or small vessel bleeding (20%) [13].

Venous bleeding, typically from gastroesophageal varices, is rarer and generates less catastrophic consequences [21,22].

### 3.4. Pseudoaneurysms

The pseudoaneurysms may potentially involve all arteries surrounding the pancreatic gland [22]. The time required for a pseudoaneurysm to develop may be extremely variable [13,23,24]. This is why they may complicate acute and chronic pancreatitis [25,26]. Mortality associated with hemorrhage from a ruptured pseudoaneurysm appears to be higher in patients with acute pancreatitis compared to those with chronic pancreatitis [22,25,26].

The most frequent arteries where pseudoaneurysms can form are as follows:

- Splenic artery (35–50%);
- Gastroduodenal and pancreaticoduodenal arteries (20–25%);
- Mesenteric, colic, and hepatic arteries (5%);
- Aorta (0.5%) [13,22].

### 3.5. Hemorrhagic Pancreatic Collection

Pancreatic collections, such as pseudocysts and walled-off necrosis (WON), are a local complication of acute pancreatitis, and they usually take about four weeks to develop. These collections are characterized by a well-defined and vascularized wall of reactive tissue surrounding the fluid (i.e., pseudocyst) or necrotic (i.e., WON) content [4,6]. The increase in their volume together with the increasing pressure that develops inside these collections may lead to necrosis of the surrounding capsule and, consequently, to its disruption. This may cause active bleeding from the capsule itself. Moreover, the leak of pancreatic content from the collection into the highly vascularized retroperitoneal space may lead to the erosion of surrounding vessels and subsequent hemorrhage [13]. Finally, this pressure necrosis process may also involve the surrounding viscera (e.g., duodenum), and it could lead to the spontaneous rupture of the collection into hollow viscus [21].

### 3.6. Timing of Bleeding

In these patients, the bleeding may occur early after the onset of acute pancreatitis. However, it is usually seen as a late complication (26–27 days after the onset of acute pancreatitis), and sometimes, it might even happen in patients with chronic pancreatitis [13,19,21,22].

Intraabdominal and postoperative bleeding typically manifests later in the disease course, while gastrointestinal bleeding can arise at any point [18].

### 3.7. Mortality

Although quite rare, the mortality rate secondary to bleeding events is extremely high, ranging between 30% and 50% [18,19,20,21,27].

Mortality is usually higher if the bleeding is intraabdominal compared to gastrointestinal, if it manifests lately (i.e., more than >7 days after the onset of pancreatitis), and/or if it is managed with an acute phase surgical operation [18,21].

### 3.8. Risk Factors

Many risk factors for hemorrhage in patients affected by pancreatitis have been identified:

- Pancreatic necrosis;
- Peripancreatic infection;
- Fungal sepsis;
- Disease severity and multiorgan failure;
- Vasculitis;
- Pancreatic surgery;
- Drainage of pancreatic collections;
- Long-term anticoagulation;
- Lumen-apposing metal stent [13,18,19,21,28].

## 4. Pathogenesis of Bleeding

**Figure 1:**
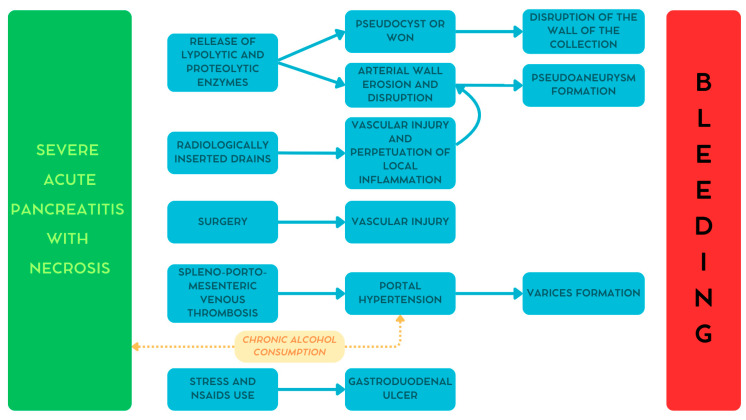
The pathogenesis of bleeding secondary to pancreatitis. WON—walled-off necrosis. Reproduced with permission from Bellio, G., et al. [29].

As reported earlier, hemorrhage in patients with pancreatitis may be caused by several conditions [13,21]. Figure 1 summarizes the different pathways involved in the development of bleeding in these patients [29].

### 4.1. Pseudoaneurysm

During pancreatitis, mainly in severe necrotic cases, lipolytic and proteolytic enzymes are released from the gland. The digestive action of these enzymes may cause progressive peripancreatic arterial wall erosion leading to the development of pseudoaneurysms [13,20,21].

### 4.2. Hemorrhagic Collection

Similar to the mechanism reported above, the pancreatic enzymes digest the retroperitoneal tissues causing the formation of peripancreatic collections. With time these collections evolve into either pseudocysts or walled-off necrosis, characterized by a well-defined and vascularized wall. The disruption of this wall may cause bleeding [13,20,21].

### 4.3. Direct Vascular Injury

Peripancreatic collections may require the insertion of radiologically or endoscopically guided drains or, in advanced cases, even surgery. These invasive procedures may cause direct injury to the peripancreatic vessels. Moreover, the prolonged use of drains may perpetuate local inflammation leading to pseudoaneurysm formation [13,21,30].

### 4.4. Varices

One of the most common vascular complications during pancreatitis is the onset of venous thrombosis along the portal system. In severe cases, this condition can lead to portal hypertension, causing in turn the formation of gastroesophageal varices. Additionally, chronic alcohol consumption, one of the main causative agents for pancreatitis, may contribute to the development of cirrhosis and, consequently, portal hypertension and varices formation [20,31].

### 4.5. Gastroduodenal Ulcer

One more possible cause of bleeding, although rare, is gastroduodenal ulcer. In fact, patients with pancreatitis might assume non-steroidal anti-inflammatory drugs that, together with the increased stress due to pancreatitis itself, may increase the risk of developing a peptic ulcer. Even in this case, chronic alcohol consumption may play a relevant role [18,20,30].

### 4.6. Classification

Bleeding secondary to pancreatitis may be classified according to the following:

- Origin: arterial or venous;
- Site: intraluminal (i.e., gastrointestinal) or extraluminal (i.e., intraabdominal);
- Correlation: spontaneous or post-procedural (i.e., postoperative or post-drainage);
- Severity: minor or major (i.e., acute reduction in Hb level of at least 2 g/dL and/or new onset of hemodynamic instability);
- Timing: early or late (the cut-off is 7 days from the onset of acute pancreatitis) [18,21].

Characterizing the type of bleeding is important to determine the best strategy to approach and treat it [21].

## 5. Clinical Presentation

The correct and timely diagnosis of ongoing abdominal bleeding in acute pancreatitis is a crucial step for planning subsequent treatment to prevent potentially life-threatening complications.

### Symptoms and Signs

The correct interpretation of signs and symptoms is the first step in diagnostic suspicion. Massive acute bleeding can present as hemorrhagic shock associated with intense abdominal pain. The patient may appear hypotensive, tachycardic, restless, or tachypneic, with pale skin and sometimes mottling, and weak peripheral pulses. Laboratory measurements of cellular hypoperfusion include base deficit and lactate levels obtained from blood gas analysis [32,33]. Other useful laboratory values in a patient with severe bleeding include hemoglobin and international normalized ratio (INR), which can help predict the need for massive transfusion. Platelet count and fibrinogen levels should be measured and normalized. Coagulopathy should also be identified, and resuscitation with blood products should be guided by monitoring clot formation kinetics using viscoelastic tests such as thromboelastography or rotational thromboelastometry. These laboratory measurements indicate the severity of shock, the need to mobilize blood bank resources, and the presence and type of coagulopathy [32]. According to Gupta et al. [21], the severity of hemorrhage from acute pancreatitis can be divided into the following:

- Major bleeding: acute loss of 2 g/dL of hemoglobin in cases of overt bleeding or the onset of hemodynamic instability in an otherwise stable patient, once other causes of shock, such as septic shock, are excluded;
- Minor bleeding: bleeding that does not meet the criteria for major bleeding.

Historically, the objective signs of Cullen and Grey-Turner, represented by periumbilical and flank ecchymoses, respectively, were considered specific for hemorrhagic necrotizing acute pancreatitis [34]. Both signs are actually secondary to retroperitoneal bleeding, with blood and necrotic material leaking through the fascial planes. The Grey-Turner sign represents the direct passage of hemorrhagic retroperitoneal material from the pararenal space to the quadratus lumborum muscle and the transversalis fascia. The Cullen sign represents passage through the gastro-hepatic ligament toward the falciform ligament and the periumbilical region [34,35]. Despite being well-known, recent studies have shown that both signs have low sensitivity in diagnosing hemorrhagic acute pancreatitis (appearing in <1% of patients); however, their presence indicates retroperitoneal hemorrhage and is associated with high mortality (50–70%) [36].

Intraluminal bleeding may present with general hemorrhagic symptoms and signs, as well as typical signs of acute gastrointestinal tract bleeding, including hematemesis (or blood loss from the nasogastric tube), enterorrhagia, and melena [21,37].

## 6. Imaging

### 6.1. Ultrasound

In cases of intraabdominal bleeding, free fluid may be seen on focused assessment with sonography for trauma (FAST) ultrasound, although it may be difficult to detect due to the preexisting inflammatory process. In experienced hands, Doppler ultrasound can be helpful in recognizing vascular complications, such as pseudoaneurysms of large vessels like the splenic artery [38]. This method is less useful in cases of retroperitoneal and intraluminal bleeding.

### 6.2. Computed Tomography (CT) Scan

**Figure 2:**
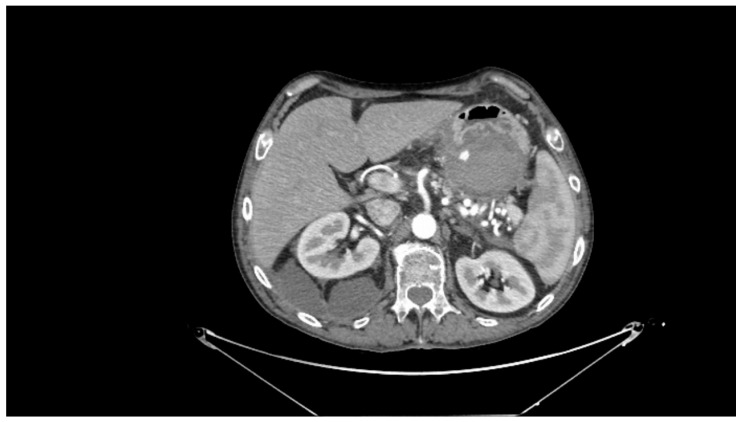
Contrast-enhanced abdominal computed tomography showing active bleeding inside a peripancreatic collection. Reproduced with permission from Bellio, G., et al. [29].

Contrast-enhanced CT scan remains a fundamental study for diagnosing complications of pancreatitis, including bleeding [31]. In cases of acute hemorrhagic pancreatitis, unenhanced CT typically shows significant enlargement of the pancreas with areas of low attenuation. After intravenous contrast administration, the pancreas displays irregular contrast enhancement, indicating areas of normal perfusion (i.e., viable parenchyma) and regions of reduced or abnormal perfusion (i.e., suggesting edema or necrosis) [21,31,39]. In cases of direct vascular damage or intraluminal bleeding, extravasation of contrast material can be seen, a highly specific finding for active bleeding (Figure 2) [31,40].

### 6.3. Angiography

Selective angiography is the standard diagnostic tool for abdominal bleeding secondary to pancreatitis, highlighting contrast extravasation from blood vessels, with a sensitivity of up to 100%. This method allows us to accurately assess collateral circulation and determine the precise vessel or site of bleeding, both important elements for planning the most appropriate subsequent treatment. The main drawback of using this imaging modality for making a diagnosis is its invasiveness and, consequently, the not negligible complication rate related, such as access site hematomas, femoral artery pseudoaneurysms, thrombosis, and/or dissection [40]. Anyhow, angiography is useful not only for diagnosis but also for potential therapeutic interventions via interventional radiology [13,40,41].

### 6.4. Endoscopy

In cases of suspected intraluminal bleeding, endoscopy of the upper gastrointestinal tract should be considered the method of choice for diagnosing the bleeding site, due to its high diagnostic sensitivity and specificity, as well as its therapeutic potential. This option is suitable for hemodynamically stable patients. If endoscopic diagnosis fails or the patient is hemodynamically unstable, a CT scan with contrast should be the next choice [21].

## 7. Treatment

**Figure 3:**
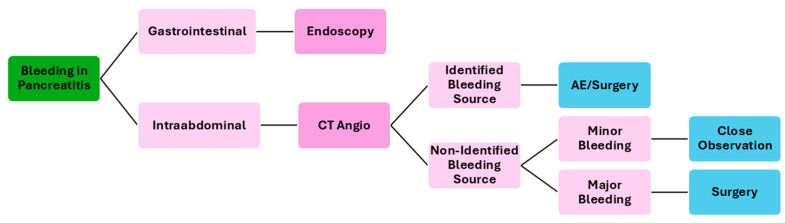
Flowchart of the management of bleeding patients secondary to pancreatitis. CT—computed tomography; AE—angioembolization.

The treatment of acute bleeding secondary to pancreatitis is multifaceted and involves a combination of endoscopic, interventional radiology, surgical, and medical approaches, tailored to the patient’s specific condition and the severity of the bleeding. Figure 3 resumes a possible flowchart for the management of bleeding patients with pancreatitis.

### 7.1. Medical Management and Supportive Care

Rapid fluid resuscitation is crucial for managing shock and ensuring adequate organ perfusion, often with crystalloids and blood products [42]. For patients with refractory hypotension, vasoactive agents like norepinephrine can be used to maintain blood pressure.

Effective analgesia is essential, non-steroidal anti-inflammatory drugs (NSAIDs), and opioids are the most frequently prescribed analgesics for pain [42]. However, careful dosing is necessary to avoid adverse effects on pancreatic function and gastrointestinal motility. It is possible to use a multimodal analgesic strategy in order to reach pain control, and thoracic epidural analgesia may also be considered in severe intractable pain.

Fresh frozen plasma or platelets may be used to reduce bleeding risk.

### 7.2. Endoscopic Management

Endoscopic hemostasis is effective for controlling localized, accessible bleeding such as gastroesophageal varices and ulcers. Techniques such as endoscopic clipping, thermal coagulation, and band ligation may allow control of the bleeding [21].

Endoscopy is a useful tool for the simultaneous identification and treatment of bleeding sources.

### 7.3. Interventional Radiology Techniques—Angiographic Embolization

Transcatheter arterial embolization (TAE) is commonly the first-choice treatment for active bleeding in hemodynamically stable or resuscitated patients with pancreatitis. The splenic artery is the most common location of pseudoaneurysm (35–50%), but other arteries may be involved such as the gastroduodenal, pancreaticoduodenal, hepatic, or left gastric arteries [13,26,43]. The procedure typically involves accessing the femoral artery to guide catheters to the bleeding site. The choice of embolic material varies depending on many factors like the artery involved, pseudoaneurysm characteristics, and patient condition, one or a combination of two or more agents is often used. Coils are usually the first agent of choice offering excellent control. Hemostasis can be increased by thrombin, gelfoam, N-butyl cyanoacrylate glue, or other materials [13,44].

This minimally invasive option can stabilize patients and often allows faster recovery and repeatability if needed. However, it may not be suitable for particularly large pseudoaneurysms or situations where the bleeding vessel cannot be accessed. It is important to emphasize the risk of rebleeding (17–37%) and the potential need for repeated interventions [20,43,44].

In highly specialized centers, TAE may be performed for hemodynamically unstable patients experiencing severe bleeding, such as in cases of splanchnic artery pseudoaneurysms (SAPA) or gastrointestinal hemorrhage unresponsive to endoscopic interventions. In patients with hemodynamic instability due to SAPA, TAE has demonstrated a high success rate (94%) in controlling bleeding and restoring stability [45]. For gastrointestinal hemorrhages, super-selective TAE serves as an emergency option to stop persistent bleeding, achieving a technical success rate of 98% with low ischemic complications, thus providing a viable alternative to surgical intervention in high-risk settings [46].

### 7.4. Surgical Intervention

Surgery is typically reserved for cases where endoscopic and angiographic techniques have failed or for patients with ongoing hemodynamic instability or large pseudoaneurysms (over 10 cm). Surgery may also be necessary for managing infected or necrotic pancreatic tissue [42,43].

Surgical options can include necrosectomy (minimally invasive or open), partial pancreatectomy, vessel ligation, or drainage of hemorrhagic fluid collections. Since pseudoaneurysms are a common cause of bleeding in chronic pancreatitis, surgical management may involve resection of these lesions, often along with the management of pancreatic pseudocysts [20,43]. However, surgery carries significant risks, particularly for critically ill patients.

In case of venous bleeding, particularly when it is diffuse, the only treatment possible is damage control surgery such as retroperitoneal packing with moist gauze. The wound packs should remain moist and be gently removed when medication is administered [20].

Surgery is associated with high rates of postoperative complications, including pancreatic fistula formation (10–30%), intraabdominal abscess (15–25%), and wound infections (10–20%), and, in severe cases, postoperative bleeding (5–10%) and multiorgan failure (10–20%) [44]. Management of these complications may involve a multidisciplinary approach, such as drainage of fistulas and percutaneous drainage of intraabdominal abscesses. Postoperative management includes vigilant monitoring for signs of complications, early intervention when they occur, and supportive care tailored to the patient’s needs. Nutritional support, infection control, and management of organ dysfunction are integral components of postoperative care, as these patients can present with higher rates of infections or nutritional deficiencies.

## 8. Preventive Strategies

Prevention relies heavily on the early and aggressive management of pancreatitis, close monitoring for vascular complications, and, where appropriate, selective prophylactic interventions; a multidisciplinary approach is essential for optimizing patient outcomes.

### 8.1. Early Pancreatitis Management

Prompt management of the underlying pancreatitis can help reduce complications, including bleeding. This includes adequate fluid resuscitation, pain control, and nutritional support to prevent pancreatic necrosis, which is a major risk factor for bleeding [42].

### 8.2. Control of Pancreatic Infections

In cases of sterile necrosis, a too-aggressive approach should be avoided to prevent further trauma, where the use of soft drains, and careful positioning of these drains away from big vessels, is suggested [20,43]. However, for infected necrosis, debridement of septic foci is critical to prevent vascular erosion and bleeding [44]. Infection control through antibiotics or drainage of infected pancreatic fluid collections can prevent bleeding and other severe complications.

### 8.3. Monitoring for Vascular Complications

Patients with severe or necrotizing pancreatitis should be closely monitored for signs of vascular complications, such as pseudoaneurysm formation or splenic vein thrombosis, through imaging (i.e., CT, MRI, or Doppler ultrasound). Early detection allows for timely intervention, potentially preventing a bleeding episode [20,23,31].

### 8.4. Prophylactic Angioembolization

In selected high-risk patients (e.g., those with pseudoaneurysms), prophylactic angioembolization can be considered to prevent rupture and subsequent hemorrhage [43].

### 8.5. Anticoagulants and NSAIDs

The pro-thrombotic state associated with acute pancreatitis and the use of anticoagulant therapy to enhance microcirculation could potentially improve patient outcomes. Although there has been debate about the initiation of chemoprophylaxis, recent studies indicate that antithrombotic prophylaxis during acute pancreatitis does not appear to significantly raise bleeding risk. Additionally, its effectiveness in reducing thrombotic complications, including but not limited to splanchnic thrombosis, supports its potential use [47]. However, in patients with a high bleeding risk, the decision to use anticoagulants and NSAIDs should be carefully weighed, as these may increase bleeding risk. For these patients, alternative approaches for pain management and anticoagulation may be necessary.

## 9. Conclusions

Hemorrhage, although infrequent, is a significant complication of pancreatitis, associated with substantial morbidity and mortality. Patients with necrotizing pancreatitis, particularly those with infection, are at increased risk. Therefore, a high level of clinical suspicion is warranted. Early diagnosis and expeditious management are essential to optimize patient outcomes.

## Data Availability

No new data were created or analyzed in this study.
